# Anti-Inflammatory Activity of 4-(4-(Heptyloxy)phenyl)-2,4-dihydro-3*H*-1,2,4-triazol-3-one via Repression of MAPK/NF-κB Signaling Pathways in β-Amyloid-Induced Alzheimer’s Disease Models

**DOI:** 10.3390/molecules27155035

**Published:** 2022-08-08

**Authors:** Fengmao An, Xinran Xuan, Zheng Liu, Ming Bian, Qingkun Shen, Zheshan Quan, Guowei Zhang, Chengxi Wei

**Affiliations:** 1 Institute of Pharmaceutical Chemistry and Pharmacology, Inner Mongolia Minzu University, Tongliao 028000, China; 2 Inner Mongolia Key Laboratory of Mongolian Medicine Pharmacology for Cardio-Cerebral Vascular System, Tongliao 028000, China; 3 Medical College, Inner Mongolia Minzu University, Tongliao 028000, China; 4 First Clinical Medical College, Inner Mongolia Minzu University, Tongliao 028000, China; 5 College of Pharmacy, Yanbian University, Yanji 133002, China; 6 College of Nursing, Inner Mongolia Minzu University, Tongliao 028000, China; 7 Institute of Dementia, Inner Mongolia Minzu University, Tongliao 028000, China

**Keywords:** triazoles, Alzheimer’s disease, amyloid beta-peptides, neuroinflammation, mitogen-activated protein kinase/NF-kappa B signaling pathways

## Abstract

Alzheimer’s disease (AD) is a major neurodegenerative disease, but so far, it can only be treated symptomatically rather than changing the process of the disease. Recently, triazoles and their derivatives have been shown to have potential for the treatment of AD. In this study, the neuroprotective effects of 4-(4-(heptyloxy)phenyl)-2,4-dihydro-3*H*-1,2,4-triazol-3-one (W112) against β-amyloid (Aβ)-induced AD pathology and its possible mechanism were explored both in vitro and in vivo. The results showed that W112 exhibits a neuroprotective role against Aβ-induced cytotoxicity in PC12 cells and improves the learning and memory abilities of Aβ-induced AD-like rats. In addition, the assays of the protein expression revealed that W112 reversed tau hyperphosphorylation and reduced the production of proinflammatory cytokines, tumor necrosis factor-α and interleukin-6, both in vitro and in vivo studies. Further study indicated that the regulation of mitogen-activated protein kinase/nuclear factor-κB pathways played a key role in mediating the neuroprotective effects of W112 against AD-like pathology. W112 may become a potential drug for AD intervention.

## 1. Introduction

Alzheimer’s disease (AD) is a complex neurodegenerative disorder with clinical characteristics including memory loss, dementia, and cognitive impairment [1], and represents a very important public healthcare problem with a serious economic burden for the society [2]. There are many contributing factors for AD. The main pathological features of AD include extracellular senile plaques (SPs) containing amyloid beta (Aβ), intracellular neurofibrillary tangles (NFTs) composed of hyperphosphorylated tau protein, the loss of synaptic and neuronal function, and neuronal death [3]. The causes for the vast majority of AD cases are unknown and satisfactory therapeutic and preventive measures for AD are unavailable.

The abnormal and excessive production, accumulation, and aggregation of Aβ is regarded as important causal factors in the pathogenesis of AD. Aβ is a peptide of 36–43 amino acid residues that results from β- and γ-secretase-mediated cleavage of transmembrane amyloid precursor proteins (APP) [4]. The deposition of Aβ is considered to occur decades before significant decline in brain cognitive abilities. The hyperphosphorylation of tau protein is another pathological manifestation of AD. Tau is a microtubule-associated protein and participates in maintaining microtubule assembly and stabilization, particularly in the axon. The hyperphosphorylated tau forms NFTs causing synapses loss, axonal transport impairment, mitochondrial dysfunction, and finally neuronal death and neurodegeneration [5]. The amyloid-cascade hypothesis posits that Aβ accumulation drives tau pathology and tau-mediated neurodegeneration in AD. Direct interaction of Aβ_1–42_ promoting the tau aggregation and hyperphosphorylation has been reported [6]. However, the precise mechanism behind how Aβ induces neurodegeneration and cognitive decline in AD remains unclear. This hypothesis has guided most drug discovery efforts in AD; however, they have not been successful in slowing cognitive decline in AD patients. A growing body of evidence suggests that inflammation plays an important role in AD pathogenesis. Both the aggregation of Aβ and the hyperphosphorylation of tau are accompanied by inflammation [7,8]. In AD, the excessive Aβ production and the hyperphosphorylated tau dysregulate the immune clearance mechanism. Cytokines, including tumor necrosis factor-α (TNF-α) and interleukin-6 (IL-6), are increased under the action of Aβ and the hyperphosphorylated tau. Furthermore, the major pro-inflammatory cytokines are closely related to mitogen-activated protein kinase (MAPK)/nuclear factor-κB (NF-κB) which signals activation of pathways and expands the chronic inflammatory response. 

In recent years, triazoles and their derivatives have received significant interest due to their pharmacological importance [9]. Triazoles are five-membered rings, which contain two carbon and three nitrogen atoms. According to the position of nitrogen atoms, the triazoles exist in two tautomeric forms, the 1,2,3-triazole and the 1,2,4-triazole. Both isomeric forms and their derivatives provide an effective approach for the treatment of many neurological disorders such as epilepsy. Our co-workers designed and synthesized several series of triazole derivatives with anticonvulsant activity [10,11]. Among them, 4-(4-(heptyloxy)phenyl)-2,4-dihydro-3*H*-1,2,4-triazol-3-one (W112) has attracted our interest due to its low neurotoxicity and valuable antiepileptic activity [12] (original Patent No. CN 102824342 A). Recently, triazole-based compounds are under study for the treatment of neurodegenerative disease, including AD. Most of the synthesized compounds displayed potential acetylcholinesterase inhibitory activity for the treatment of AD [13,14]. In addition, Kaur et al. designed and synthesized multi-target-directed triazole derivatives which could inhibit Aβ_42_ aggregation and the reactive oxygen species production [15]. The findings of Fronza et al. support that QTC-4-MeOBnE could reverse neuronal loss, reduce oxidative stress, and ameliorate synaptic function through Na+/K+ ATPase and acetylcholinesterase activities in an STZ-induced mouse model [16]. However, the in vivo and in vitro studies and related mechanisms of triazoles against AD pathology still need to be further explored. Therefore, the aim of the current experiment is to investigate the effect of W112 on AD-like pathology induced by Aβ_25–35_ in vivo and in vitro as well as the molecular mechanism involved.

## 2. Results

### 2.1. Effects of W112 on Cell Survival in Aβ_25–35_-Induced PC12 Cells

**Figure 1:**
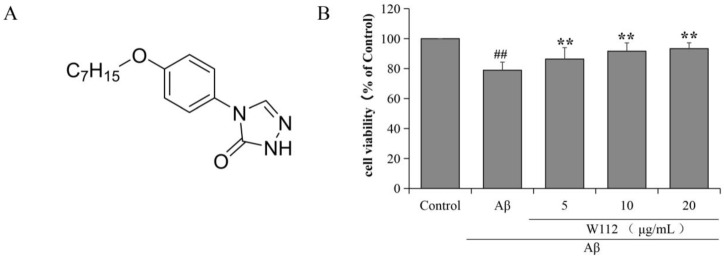
The chemical structure of W112 and the effect of W112 on the viability of PC12 cells induced by Aβ_25–35_. (**A**) Chemical structure of W112. (**B**) The MTT assay was used to detect the effect of W112 on PC12 cells activity. *n* = 6. ^##^ *p* < 0.01 vs. the control group; ** *p* < 0.01 vs. the model group.

To evaluate the cytotoxicity of Aβ_25–35_ and explore the neuroprotection of W112 treatment, we first used Aβ_25–35_ to injure PC12 cells in the absence and presence of W112 for 48 h and cell vitality was detected by MTT assay. The results in Figure 1 showed that Aβ treatment markedly decreased cell viability (*p* < 0.01), while the cells treated with 5, 10, and 20 μg/mL W112 significantly exhibited higher viability than the models (*p* < 0.01). These results indicate that W112 exhibits a neuroprotective role against Aβ-induced cytotoxicity.

### 2.2. Effects of W112 on Spatial Learning and Memory Abilities in Rats

**Figure 2:**
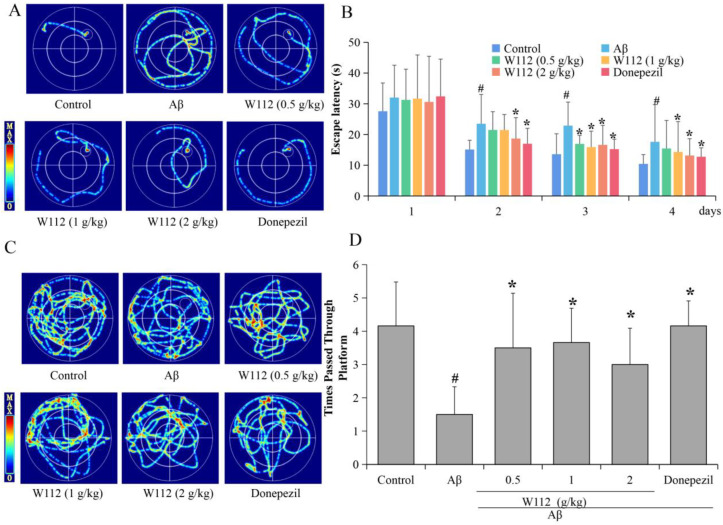
W112 treatment improved cognitive impairments of AD rats. (**A**,**B**) The representative swim paths and the escape latency. (**C**,**D**) The representative swim paths and the number of platform crossings. *n* = 12. ^#^ *p* < 0.05 vs. the control group; * *p* < 0.05 vs. the model group.

To evaluate the spatial learning and memory ability of Aβ_25–35_-induced AD rats and the protective effects of W112, the Morris water maze (MWM) test was executed. Figure 2A,B showed that in the place navigation test, the escape latency time of the model group was significantly longer than that of the control group (*p* < 0.05). Compared with the model group, the escape latency time of W112 groups was significantly shorter (*p* < 0.05), indicating that W112 could improve spatial learning ability. In the probe test, the numbers crossing the platform in the model group were significantly less than those in the control group (*p* < 0.05), and the numbers crossing the platform in W112 groups were significantly higher than those in the model group (*p* < 0.05), indicating that W112 could improve cognition (Figure 2C,D).

### 2.3. Effects of W112 on Aβ_25–35_-Induced Tau Hyperphosphorylation

**Figure 3:**
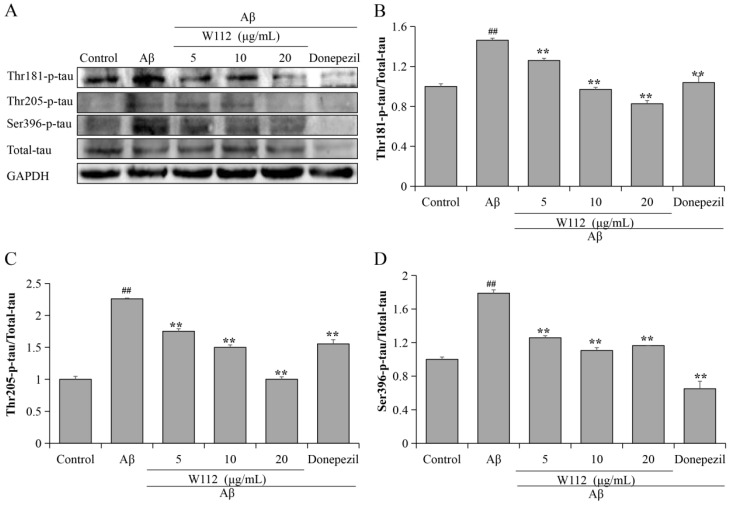
W112 prevented tau pathology in Aβ_25–35_-induced PC12 cells. (**A**–**D**) The protein levels of phosphorylated tau at thr181, thr205, and Ser396 sites in PC12 cells were measured by Western blot. *n* = 3. ^##^ *p* < 0.01 vs. the control group; ** *p* < 0.01 vs. the model group.

**Figure 4:**
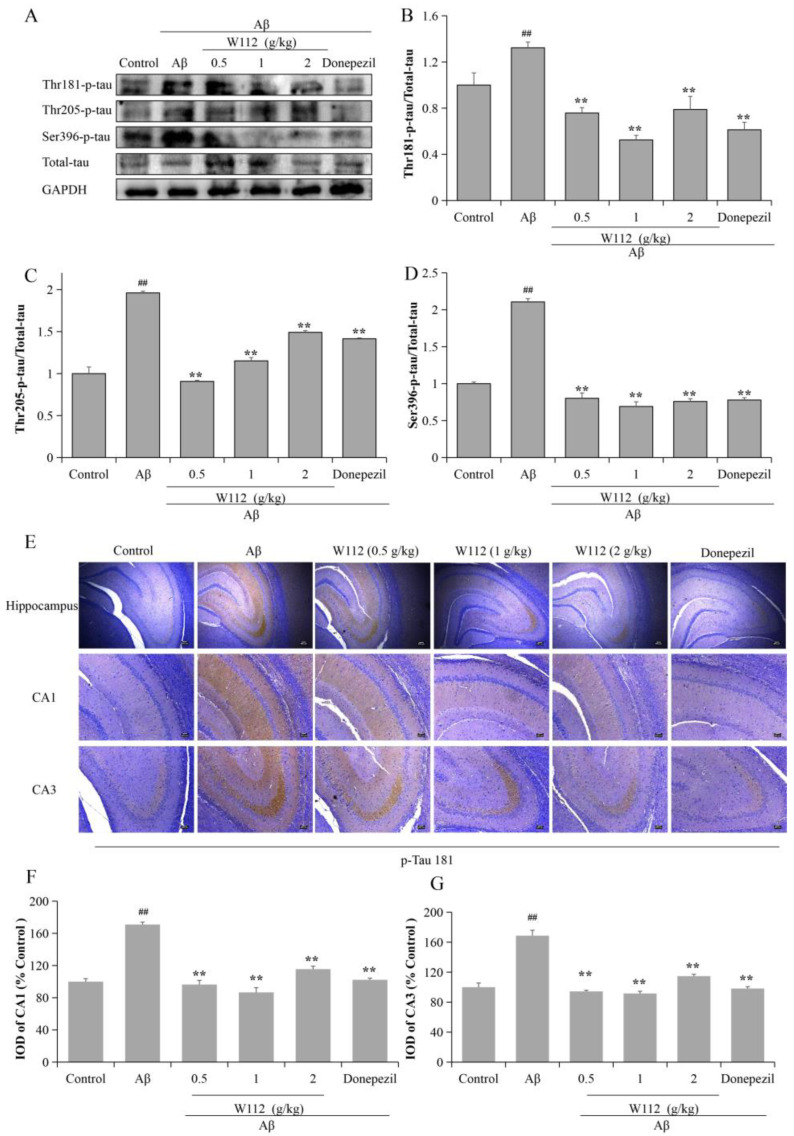
W112 prevented tau pathology in Aβ_25–35_-induced rat model. (**A**–**D**) The protein levels of phosphorylated tau at thr181, thr205, and Ser396 sites in the hippocampus of rat model were measured by Western blot. *n* = 8. (**E**–**G**) IHC staining of rat brain immunostained with antibodies against thr181-phosphorylated tau (scale bar, Hippocampus: 500 µm; CA1, CA3 region: 250 µm). *n* = 4. ^##^ *p* < 0.01 vs. the control group; ** *p* < 0.01 vs. the model group.

The tau protein is a principle neuropathological hallmark of AD. The hyperphosphorylation of tau protein seriously damages the microtubule structure and affects the synthesis, release, and transport of neurotransmitters, and eventually leads to the occurrence of AD. In the present study, we evaluated the effects of W112 on tau hyperphosphorylation both in vivo and in vitro via Western blot. Figure 3A–D showed that the levels of phosphorylated tau at thr181, thr205, and Ser396 sites were significantly higher in the Aβ_25–35_-induced cell model (*p* < 0.01), while W112 treatment reduced the levels of tau hyperphosphorylation (*p* < 0.01). Furthermore, in vivo*,* as shown by the results in Figure 4A–D, W112 treatment also reduced the levels of phosphorylated tau at multiple sites induced by Aβ_25–35_ in the hippocampus of a rat model (*p* < 0.01). Based on the rat model, we further used an immunohistochemistry (IHC) assay to detect the level of tau phosphorylation at the thr181 site in the hippocampus. Positive staining of phosphorylated tau was significantly decreased after W112 treatment in the hippocampus (Figure 4E). The results displayed that W112 could significantly prevent tau pathology in Aβ_25–35_-induced cell and rat models.

### 2.4. Effects of W112 on the Aβ_25–35_-Induced Neuroinflammation

**Figure 5:**
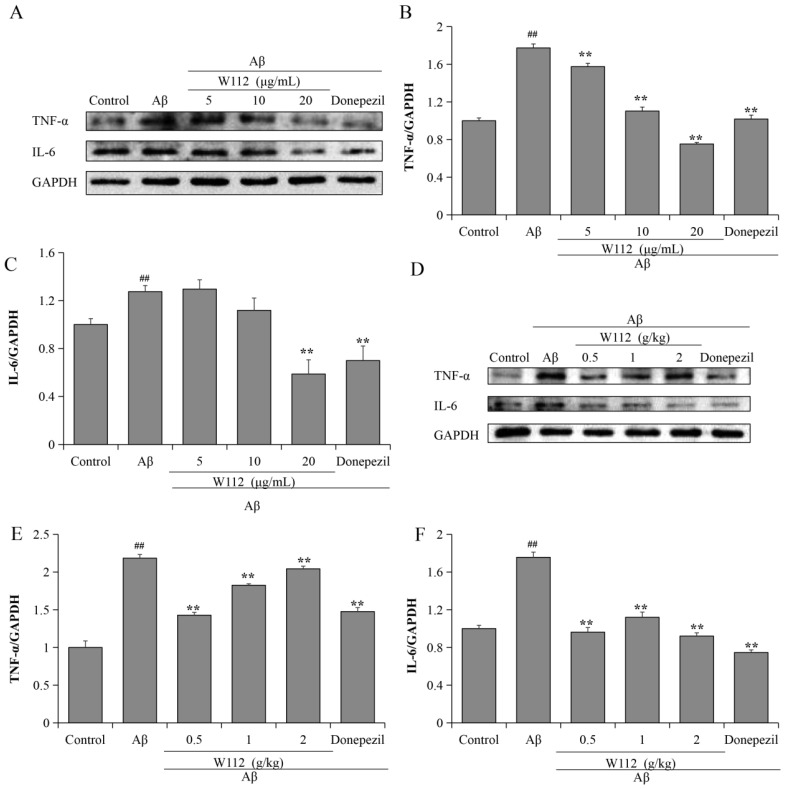
W112 suppressed the production of proinflammatory cytokines TNF-α and IL-6 in Aβ_25–35_-induced cell and rat models. (**A**–**C**) The expressions of TNF-α and IL-6 in PC12 cells were measured by Western blot. *n* = 3. (**D**–**F**) The expressions of TNF-α and IL-6 in the hippocampus were measured by Western blot. *n* = 8. ^##^ *p* < 0.01 vs. the control group; ** *p* < 0.01 vs. the model group.

To investigate the anti-neuroinflammatory activity of W112, we detected classic inflammation-related factors, such as TNF-α and IL-6, via Western blot. Compared with the model group, treatment with Aβ_25–35_ significantly increased the expression of TNF-α and IL-6, while W112 markedly suppressed the production of TNF-α and IL-6 both in vitro (*p* < 0.01; Figure 5A–C) and in vivo (*p* < 0.01; Figure 5D–F). 

### 2.5. Effects of W112 on Inhibition of NF-κB Signaling Pathway

**Figure 6:**
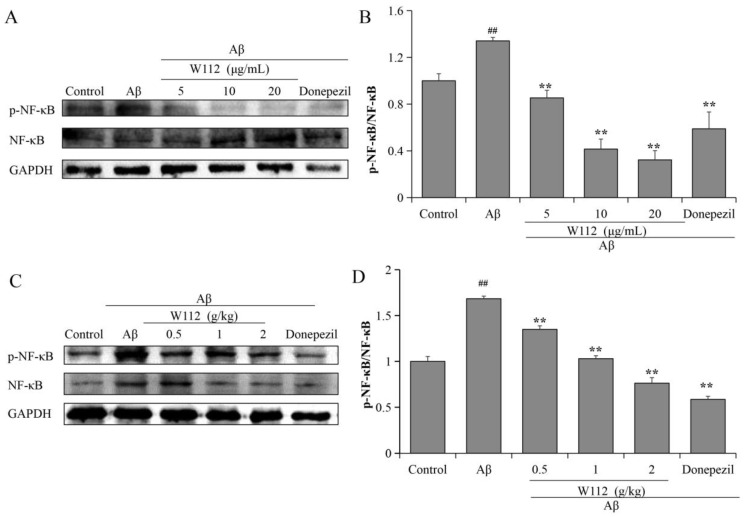
W112 treatment suppressed the phosphorylation of NF-κB signaling pathway in Aβ_25–35_-induced cell and rat models. (**A**,**B**) The phosphorylation and total levels of NF-κB in PC12 cells were measured by Western blot. *n* = 3. (**C**,**D**) The phosphorylation and total levels of NF-κB in the hippocampus were measured by Western blot. *n* = 8. ^##^ *p* < 0.01 vs. the control group; ** *p* < 0.01 vs. the model group.

To assess the effects of W112 on inhibition of NF-κB signaling, we evaluated the level of p-NF-κB/NF-κB by Western blot assay. The level of p-NF-κB/NF-κB in the model group was markedly higher compared with the control group, while W112 treatment significantly decreased p-NF-κB/NF-κB level both in vitro (*p* < 0.01; Figure 6A,B) and in vivo (*p* < 0.01; Figure 6C,D), suggesting that W112 treatment suppressed the phosphorylation of NF-κB signaling.

### 2.6. Effects of W112 on MAPK Signaling Pathway

**Figure 7:**
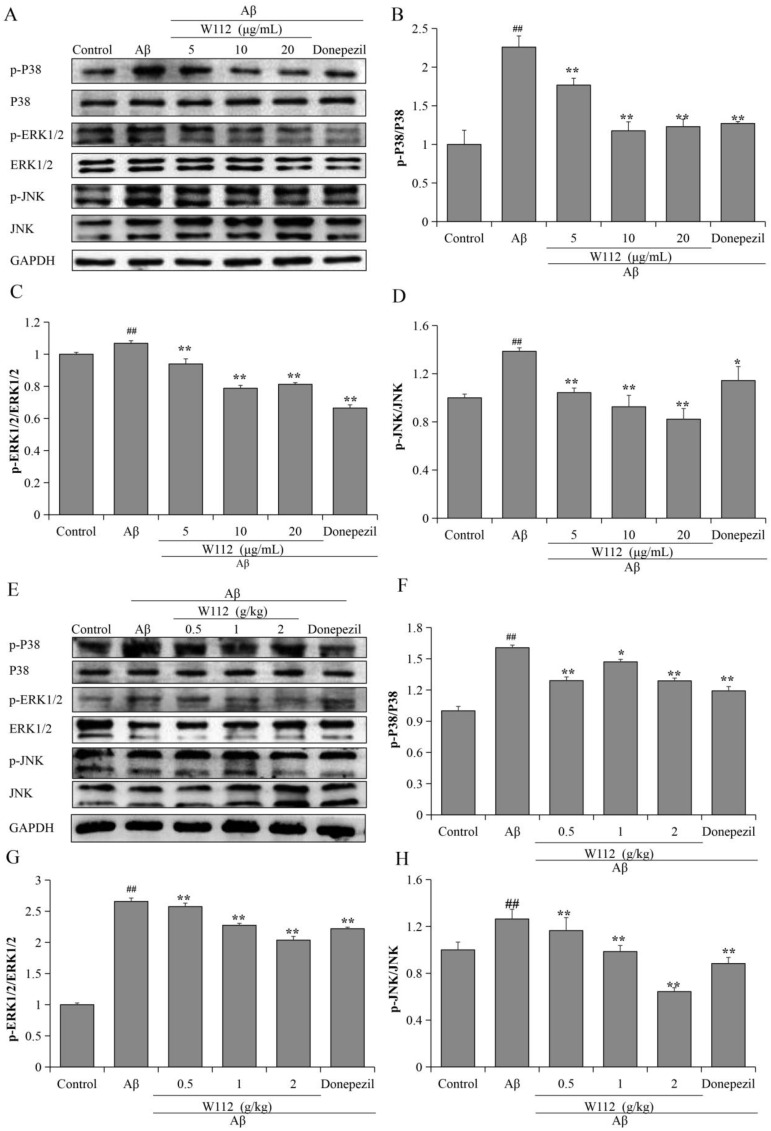
W112 treatment inhibited the activation of MAPK signaling pathway in Aβ_25–35_-induced cell and rat models. (**A**–**D**) The phosphorylation and total levels of p38, ERK1/2, and JNK in PC12 cells were measured by Western blot. *n* = 3. (**E**–**H**) The phosphorylation and total levels of p38, ERK1/2, and JNK in the hippocampus were measured by Western blot. *n* = 8. ^##^ *p* < 0.01 vs. the control group; * *p* < 0.05, ** *p* < 0.01 vs. the model group.

To further investigate the molecular mechanisms of W112-mediated intervention of Aβ_25–35_-induced AD-like pathology, we evaluated the effects of W112 on MAPK signaling by Western blot assay. The phosphorylation levels of p38, extracellular signal-regulated kinases 1/2 (ERK1/2), and c-Jun N-terminal kinase (JNK) were significantly up-regulated by Aβ_25–35_ compared with the control group, while W112 effectively reduced the abnormal ratios of p-p38/p38, p-ERK1/2/ERK1/2, and p-JNK/JNK both in vitro (*p* < 0.05 or *p* < 0.01; Figure 7A–D) and in vivo (*p* < 0.05 or *p* < 0.01; Figure 7E–H).

## 3. Discussion

With global aging, the prevalence of clinical AD is 2–3 times higher every 10 years. Rajan et al. reported that, starting in 2022, the number of people aged 75–84 suffering from AD will exceed those aged 85 and over [17]. With the change of the population burden of clinical AD, this will bring greater social, personal, and economic pressure to families and societies. The pathogenesis of AD is still inconclusive, and there are many hypotheses, which are very complex. Only four drugs are commonly used to treat AD, three of which are cholinesterase inhibitors (including donepezil, galantamine, and rivastigmine) and one is memantine, a non-competitive N-methyl-D-aspartic acid (NMDA) receptor antagonist. These drugs have been shown to be effective only for mild to moderate AD, which makes treatment options for AD very constrained. In 2021, aducanumab was approved by the FDA as the first anti-amyloid monoclonal antibody. Aducanumab was reported to cross the blood brain barrier (BBB) and then selectively bind with Aβ aggregates [18]. Although aducanumab has received both praise and criticism since its approval, its approval at least gives hope for AD drug development. 

Triazoles have attracted more and more attention because of their wide range of biological activities. Triazole-based compounds are now under study for the treatment of a variety of central nervous system (CNS) diseases. The study of Gitto et al. revealed 1,2,4-triazole-based compounds could inhibit α-syn aggregation to prevent Parkinson’s disease [19]. Wu et al. worked on 1,2,4-triazole derivatives and found a series of anticonvulsant compounds by using epilepsy models during both in vivo and in vitro studies [20]. Recently, triazole-based compounds have been explored for the possibility of treating AD. Wang et al. synthesized a series of novel triazole derivatives as a multi-functional agent for AD therapy and determined that the compounds demonstrate multiple effects including anti-neuroinflammation, selective inhibition of cholinesterase, and neuroprotection [21]. In the current experiment, we mainly focused on the effects of W112 on Aβ_25–35_-induced AD-like pathological changes and the molecular mechanism. Kollmer et al. reported that brain-derived Aβ amyloid fibrils fold differs sharply from Aβ_1–40_ fibrils that were formed in vitro, and these findings underscored the importance of using patient-derived amyloid fibrils when investigating the structural basis of the disease. However, they also claimed that it would be a premature conclusion to state that in vitro formed fibrils were necessarily different from patient fibrils [22]. Millucci et al. also supported that the conditions of Aβ aggregation in the brain were different from those in the in vitro experiment and the actual aggregation kinetics would differ. However, it is very likely that this amyloid fragment also rapidly aggregated in the brain, and that changes in the actual aggregation process could result in the formation of aggregated structures that may have powerful effects on synaptic activity [23]. Therefore, in the current study, we still used humanized Aβ_25–35_ to induce aggregation and mimic the neurotoxic role, as it represents the biologically active region of Aβ_1–40_ or Aβ_1–42_ and causes enhanced neurotoxicity. We first evaluated the neuroprotective effect of W112 in the cell model and its effect on improving learning and memory abilities in the rat model induced by Aβ_25–35_. The results showed that W112 could exhibit a neuroprotective role against Aβ-induced cytotoxicity and improve the learning and memory abilities of Aβ-induced AD-like rats. These results prompted us to further study the molecular mechanisms involved. 

At present, despite the amyloid hypothesis still under investigation, it is the most mature mechanism to explain the pathogenesis of AD. The cerebral amyloid pathology appears 20–30 years earlier than the emergence of clinical AD symptoms [24]. Aβ peptides aggregate from monomers to oligomers and deposit as SPs in the extracellular, which eventually leads to the destruction of synaptic function, atrophy of neurons, and neurodegenerative changes. APP transgenic mice evidently reveal deficits in learning and memory, behavioral abnormalities, synaptic alterations, and SPs [25]. Intracellular Aβ oligomers can affect normal transmission, increase neuronal excitability of hippocampal neurons, and cause synaptic damage [26]. Mitochondria-associated membranes are an intracellular site of APP processing and Aβ is produced at mitochondria-endoplasmic reticulum contact sites, which may contribute to AD pathology [27]. Hyperphosphorylated tau represent another hallmark lesion of AD. Normal tau proteins play a vital role in neurons because of their binding and stabilizing of microtubules and regulating axonal transport. In AD pathological conditions, tau is hyperphosphorylated and aggregates into NFTs as a possible cause of memory loss and synaptic dysfunction. The microtubule-binding region of tau in cerebrospinal fluid is specifically increased and highly associated with the cognitive and clinical symptoms of AD [28]. The P301S mutant human tau transgenic mice show synaptic pathology and microglia proliferation in the hippocampus at 3 months old and synaptic dysfunction at 6 months old, which finally causes neurodegeneration [29]. The early accumulation of tau in the parietal hippocampal network is an important reason for the disorder of spatial orientation in AD [30]. The amyloid hypothesis believes that tau is a downstream target and Aβ drives tau pathology. Tau transgenic mice crossed with APP transgenic mice show that NFTs are substantially enhanced in the limbic system and olfactory cortex [31]. Aβ oligomers can cause intracellular Ca^2+^ elevation and activate the Ca^2+^-dependent calmodulin kinase IIα, which is associated with increased hyperphosphorylation and mis-sorting of tau [32]. Aβ pathology can further promote the development of tau pathology in AD by increasing the spread of pathological tau [33]. Unfortunately, despite anti-Aβ drugs reducing SPs or Aβ accumulation, most have not been shown to modify cognition in humans. Due to too many failures of anti-Aβ drug development, more and more studies are beginning to re-focus on the tau and Aβ relationship. Fá et al. reported that high concentrations of Aβ or tau alone reduced synaptic plasticity and memory, and the same result also occurred when sub-toxic doses of oligomer Aβ were used in combination with oligomer tau at sub-toxic doses [34]. Gulisano et al. supported the idea that Aβ and tau might act at the same level or on different targets, but eventually converge on a common molecular mechanism [35]. In our current study, we found that Aβ_25–35_ increased the levels of phosphorylated tau at multiple sites and W112 treatment significantly reduced the levels of tau hyperphosphorylation, both in vitro and in vivo studies. Our results (Figure 3 and Figure 4) proved that Aβ can cause tau pathology, next we tried to further explore the underlying molecular mechanisms. 

Increasing evidence shows that neuroinflammation is an active contributor to AD progression. Proinflammatory cytokines, including TNF-α and IL-6, are up-regulated in the brains of AD patients and in AD transgenic mice [36,37]. The excessive Aβ production and the hyperphosphorylated tau are both accompanied by the presence of inflammation; moreover, neuroinflammation increases the severity of the disease by exacerbating Aβ and tau pathology. Aβ-induced activation of the NLRP3 inflammasome significantly increases interleukin-1β (IL-1β) levels to enhance the progression of AD [38]. TNF-α regulates BACE-1 transcription, which results in an increased production of Aβ and further promotes TNF-α release [39]. Lipopolysaccharide injection affects inflammatory cytokine (TNF-α, IL-1β, and IL-6) production, accompanied by Aβ deposition in mouse brains [40]. The relationship between neuroinflammation and Aβ pathology is of significant concern, but few studies have paid attention to the interconnections existing between tau pathology and neuroinflammation. Neuroinflammation also plays a key role in NFTs formation. In addition, aggregated tau can further enhance inflammation and amplify neurotoxic injury. Astrocyte proliferation, microglia activation, and pathological neuroinflammation are observed in tau transgenic models [41]. Tau pathology has a direct positive correlation with neuroinflammation in the parahippocampus of AD patients examined by positron emission tomography [42]. Hyperphosphorylated tau trigger neuroinflammation in an NLRP3-dependent manner to activate IL-1β levels and impair spatial memory [43]. In this study, we found that Aβ_25–35_ could promote the release of proinflammatory cytokines, and W112 prevented the over-production of TNF-α and IL-6 both in vitro and in vivo studies. The results revealed that the mechanisms of W112 preventing the pathological process of AD may be related to the “Aβ-tau-neuroinflammation” axis.

In the nervous system, NF-κB plays an important role as a transcriptional regulator and has post-translational regulatory activity. The activation of NF-κB in the brain induces neuroinflammation, and impairs neuronal survival, differentiation, neurite growth, and synaptic plasticity, which affects the development of AD. NF-κB is activated in Aβ plaque-surrounding areas in neurons from patients with AD [44]. In Aβ-induced microglia, NF-κB was up-regulated and the production of TNF-α and IL-6 was increased [45]. Inhibition of NF-κB signaling significantly repressed neuroinflammation and ameliorated Aβ plaque load and cognitive impairment [46]. MAPK expressed in the CNS mediates neuronal proliferation, differentiation, and cell survival. The most famous MAPK enzymes are ERK1/2, JNK, and p38 families. The pathological role of MAPK cascades in AD has been reported. Aβ in the hippocampus blocked the long-term potentiation via activation of the kinases JNK and p38 [47]. P38 MAPK specifically deleted from neurons in the brain of AD transgenic mice could decrease Aβ and tau phosphorylation load and improve the cognitive function [48]. Blockade of p38, JNK, and ERK1/2 inhibited the release of TNF-α and IL-6 induced by Aβ in BV2 cells [49]. Several studies have shown that NF-κB can be activated by the MAPK pathway. MAPK regulated the transcriptional activity of NF-κB in primary human astrocytes via acetylation of p65 [50]. MAPK inhibitors can inhibit NF-κB phosphorylation and reduce TNF-stimulated IL-6 gene expression [51]. Triazole derivatives have been shown to play an anti-inflammatory role via inhibiting NF-κB activation and MAPK phosphorylation [52,53], but its effects in AD need to be further explored. In our present study, we found that W112 may potentially inhibit MAPKs/NF-κB signal pathways to reverse Aβ-induced AD-like lesions both in vitro and in vivo.

## 4. Materials and Methods

### 4.1. Preparation of Aβ_25–35_

Aβ_25–35_ (Aladdin Biochemical Technology, Shanghai, China) was dissolved in distilled water and incubated in a 37 °C incubator for 96 h to induce aggregation as previously described [54].

### 4.2. Cell Culture and Viability Assays

The PC12 rat pheochromocytoma cells were cultured in RPMI 1640 medium (Gibco, CA, USA) supplemented with 10% fetal bovine serum (FBS, Gibco) and 1% penicillin–streptomycin (Beyotime, Shanghai, China) at 37 °C in a humidified 5% CO_2_ atmosphere. Cultured cells were treated with Aβ_25–35_ (20 μmol/L) in the absence and presence of W112 (5, 10, 20 μg/mL) or donepezil (Yuanye Biological Technology Co., Ltd., Shanghai, China) as a positive control for 48 h. Cell viability was evaluated by MTT assay as previously described [47]. The absorbance of each group was measured at wavelengths of 570 nm and 630 nm.

### 4.3. Animal and Treatments

Healthy male Sprague-Dawley (SD) rats (200–220 g) were provided by Yisi Company (Changchun, China) and kept in standard laboratory conditions (temperature 23 ± 2 °C, 12-h light/dark cycles) with food and water. The rats were randomly divided into six groups (*n* = 12 in each group): control, Aβ, Aβ + W112 (0.5 g/kg bodyweight), Aβ + W112 (1 g/kg bodyweight), Aβ + W112 (2 g/kg bodyweight), and positive group (donepezil, 1 mg/kg bodyweight). The skulls of the rats were drilled with small burr holes on two sides (1.0 mm caudal to the bregma, 1.5 mm lateral to the midline). Aβ_25–35_ (15 nmol per rat) was intracerebroventricular (ICV)-injected at a depth of 3.0 mm in the Aβ, donepezil and W112 groups, and sterile normal saline was similarly injected in the control group. W112 groups received intragastric administration of 0.5, 1, and 2 g/kg W112, respectively, once daily for 28 days after the surgery. The control group and the Aβ group were treated with saline in the same way daily. The ethics approval of this study was granted by the ethical committee of the medical faculty of Inner Mongolia Minzu University (M2020015).

### 4.4. MWM Test

The MWM test was carried out under protocols detailed in previous reports [55]. The test was conducted to assess learning and memory performance. In brief, rats were trained to swim to reach the platform in a pool for 4 consecutive days and data of the escape latency were recorded. On the fifth day, the probe test was performed and the times of crossing through the original platform position were monitored by the WMT-100s Morris Water Maze video analysis system (TECHMAN, Chengdu, China).

### 4.5. Immunohistochemistry

The levels of tau phosphorylation at the thr181 site in the hippocampus from each group were detected by IHC. Briefly, the brain sections were cut at 5 µm thickness and incubated with primary antibodies against thr181-phosphorylated tau antibody (CST, Beverly, MA, USA) overnight at 4 °C. The next day, the slices were incubated with the second antibody and detected with diaminobenzidine tetrahydrochloride (Zymed, South San Francisco, CA, USA).

### 4.6. Western Blotting Analysis 

Total protein from PC12 cells or the hippocampal tissues was extracted and analyzed using Western blots. Prepared samples were separated by 10% or 12% SDS-PAGE and transferred to Polyvinylidene Fluoride (PVDF) membranes (Millpore, Bedford, MA, USA). The membranes were incubated with anti-GAPDH (Abcam, Cambridge, UK), anti-thr181-phosphorylated-tau, anti-thr205-phosphorylated-tau, anti-ser396-phosphorylated-tau, anti-total tau, anti-JNK, anti-phospho JNK, anti-ERK1/2, anti-phospho ERK1/2, anti-phospho p38, anti-p38, anti-NF-κB, anti-phospho NF-κB (CST), anti-IL-6, and anti-TNF-α (Proteintech, Wuhan, China) antibodies. Immunoreactive bands were detected with the appropriate horseradish peroxidase-conjugated secondary antibodies and immunological complexes were visualized by enhanced chemiluminescence reagents (Pierce, Rockford, IL, USA). 

### 4.7. Statistical Analysis 

Data were analyzed using SPSS 20.0 software (SPSS Inc., Chicago, IL, USA) and expressed as mean ± standard error of the mean and statistical analysis by one-way ANOVA, and the statistical significance standard was *p* < 0.05.

## 5. Conclusions

In summary, our current results showed that triazole derivative, W112, ameliorated Aβ-induced hyperphosphorylation of tau and reduced the production of proinflammatory cytokines, including TNF-α and IL-6, through significantly inhibiting MAPK/NF-κB signaling pathways both in vitro and in vivo studies. Thus, it is suggested that W112 may be a promising therapeutic strategy to prevent AD.

## Data Availability

Not available.

## Sample Availability

Samples of the compound 4-(4-(heptyloxy)phenyl)-2,4-dihydro-3*H*-1,2,4-triazol-3-one are available from the authors.

## References

[1] Lane C.A., Hardy J., Schott J.M. (2018). Alzheimer’s disease. Eur. J. Neurol..

[2] Cummings J., Aisen P.S., DuBois B., Frölich L., Jack C.R., Jones R.W., Morris J.C., Raskin J., Dowsett S.A., Scheltens P. (2016). Drug development in Alzheimer’s disease: The path to 2025. Alzheimer’s Res. Ther..

[3] Khan S., Barve K.H., Kumar M.S. (2020). Recent Advancements in Pathogenesis, Diagnostics and Treatment of Alzheimer’s Disease. Curr. Neuropharmacol..

[4] Thal D.R., Walter J., Saido T.C., Fändrich M. (2015). Neuropathology and biochemistry of Aβ and its aggregates in Alzheimer’s disease. Acta Neuropathol..

[5] Drummond E., Pires G., MacMurray C., Askenazi M., Nayak S., Bourdon M., Safar J., Ueberheide B., Wisniewski T. (2020). Phosphorylated tau interactome in the human Alzheimer’s disease brain. Brain.

[6] Rank K.B., Pauley A.M., Bhattacharya K., Wang Z., Evans D.B., Fleck T.J., Johnston J.A., Sharma S.K. (2002). Direct interaction of soluble human recombinant tau protein with Abeta 1-42 results in tau aggregation and hyperphosphorylation by tau protein kinase II. FEBS Lett..

[7] Tejera D., Mercan D., Sanchez-Caro J.M., Hanan M., Greenberg D., Soreq H., Latz E., Golenbock D., Heneka M.T. (2019). Systemic inflammation impairs microglial Aβ clearance through NLRP3 inflammasome. EMBO J..

[8] Zhang Y.H., Wang D.W., Xu S.F., Zhang S., Fan Y.G., Yang Y.Y., Guo S.Q., Wang S., Guo T., Wang Z.Y. (2018). α-Lipoic acid improves abnormal behavior by mitigation of oxidative stress, inflammation, ferroptosis, and tauopathy in P301S Tau transgenic mice. Redox Biol..

[9] Kumar S., Sharma B., Mehra V., Kumar V. (2021). Recent accomplishments on the synthetic/biological facets of pharmacologically active 1H-1,2,3-triazoles. Eur. J. Med. Chem..

[10] Liu X.J., Zhang H.J., Quan Z.S. (2017). Synthesis and evaluation of the anticonvulsant activities of 2,3-dihydrophthalazine-1,4-dione derivatives. Med. Chem. Res..

[11] Zhang G.R., Ren Y., Yin X.M., Quan Z.S. (2018). Synthesis and Evaluation of the Anticonvulsant Activities of New 5-substitued-[1,2,4]triazolo [4,3-a]quinoxalin-4(5H)-one Derivatives. Lett. Drug Des. Discov..

[12] Fang Y.Q., Sun C.L., Liu D.C., Wang S.B., Quan Z.S. (2015). Synthesis and Anticonvulsant Activity Evaluation of 3-alkoxy-4-(4-(hexyloxy/heptyloxy)phenyl)-4H-1,2,4-triazole. Iran. J. Pharm. Res..

[13] Özil M., Balaydın H.T., Şentürk M. (2019). Synthesis of 5-methyl-2,4-dihydro-3*H*-1,2,4-triazole-3-one’s aryl Schiff base derivatives and investigation of carbonic anhydrase and cholinesterase (AChE, BuChE) inhibitory properties. Bioorg. Chem..

[14] Li J.C., Zhang J., Rodrigues M.C., Ding D.J., Longo J.P., Azevedo R.B., Muehlmann L.A., Jiang C.S. (2016). Synthesis and evaluation of novel 1,2,3-triazole-based acetylcholinesterase inhibitors with neuroprotective activity. Bioorg. Med. Chem. Lett..

[15] Kaur A., Mann S., Kaur A., Priyadarshi N., Goyal B., Singhal N.K., Goyal D. (2019). Multi-target-directed triazole derivatives as promising agents for the treatment of Alzheimer’s disease. Bioorg. Chem..

[16] Fronza M.G., Baldinotti R., Sacramento M., Gutierres J., Carvalho F.B., Fernandes M.D.C., Sousa F.S.S., Seixas F.K., Collares T., Alves D. (2021). Effect of QTC-4-MeOBnE Treatment on Memory, Neurodegeneration, and Neurogenesis in a Streptozotocin-Induced Mouse Model of Alzheimer’s Disease. ACS. Chem. Neurosci..

[17] Rajan K.B., Weuve J., Barnes L.L., McAninch E.A., Wilson R.S., Evans D.A. (2021). Population estimate of people with clinical Alzheimer’s disease and mild cognitive impairment in the United States (2020–2060). Alzheimer’s Dement..

[18] Behl T., Kaur I., Sehgal A., Singh S., Sharma N., Makeen H.A., Albratty M., Alhazmi H.A., Felemban S.G., Alsubayiel A.M. (2022). “Aducanumab” making a comeback in Alzheimer’s disease: An old wine in a new bottle. Biomed. Pharmacother..

[19] Gitto R., Vittorio S., Bucolo F., Peña-Díaz S., Siracusa R., Cuzzocrea S., Ventura S., Di Paola R., De Luca L. (2022). Discovery of Neuroprotective Agents Based on a 5-(4-Pyridinyl)-1,2,4-triazole Scaffold. ACS Chem. Neurosci..

[20] Wu J., Hou Z., Wang Y., Chen L., Lian C., Meng Q., Zhang C., Li X., Huang L., Yu H. (2022). Discovery of 7-alkyloxy-[1,2,4] triazolo [1,5-a] pyrimidine derivatives as selective positive modulators of GABAA1 and GABAA4 receptors with potent antiepileptic activity. Bioorg. Chem..

[21] Wang M., Fang L., Liu T., Chen X., Zheng Y., Zhang Y., Chen S., Li Z. (2021). Discovery of 7-O-1, 2, 3-triazole hesperetin derivatives as multi-target-directed ligands against Alzheimer’s disease. Chem. Biol. Interact..

[22] Kollmer M., Close W., Funk L., Rasmussen J., Bsoul A., Schierhorn A., Schmidt M., Sigurdson C.J., Jucker M., Fändrich M. (2019). Cryo-EM structure and polymorphism of Aβ amyloid fibrils purified from Alzheimer’s brain tissue. Nat. Commun..

[23] Millucci L., Raggiaschi R., Franceschini D., Terstappen G., Santucci A. (2009). Rapid aggregation and assembly in aqueous solution of A beta (25–35) peptide. J. Biosci..

[24] Jansen W.J., Ossenkoppele R., Knol D.L., Tijms B.M., Scheltens P., Verhey F.R., Visser P.J., Aalten P., Aarsland D., Amyloid Biomarker Study Group (2015). Prevalence of cerebral amyloid pathology in persons without dementia: A meta-analysis. JAMA.

[25] Verret L., Mann E.O., Hang G.B., Barth A.M., Cobos I., Ho K., Devidze N., Masliah E., Kreitzer A.C., Mody I. (2012). Inhibitory interneuron deficit links altered network activity and cognitive dysfunction in Alzheimer model. Cell.

[26] Fernandez-Perez E.J., Muñoz B., Bascuñan D.A., Peters C., Riffo-Lepe N.O., Espinoza M.P., Morgan P.J., Filippi C., Bourboulou R., Sengupta U. (2021). Synaptic dysregulation and hyperexcitability induced by intracellular amyloid beta oligomers. Aging Cell.

[27] Schreiner B., Hedskog L., Wiehager B., Ankarcrona M. (2015). Amyloid-β peptides are generated in mitochondria-associated endoplasmic reticulum membranes. J. Alzheimer′s Dis..

[28] Horie K., Barthélemy N.R., Sato C., Bateman R.J. (2021). CSF tau microtubule binding region identifies tau tangle and clinical stages of Alzheimer’s disease. Brain.

[29] Yoshiyama Y., Higuchi M., Zhang B., Huang S.M., Iwata N., Saido T.C., Maeda J., Suhara T., Trojanowski J.Q., Lee V.M. (2007). Synapse loss and microglial activation precede tangles in a P301S tauopathy mouse model. Neuron.

[30] Stimmell A.C., Xu Z., Moseley S.C., Benthem S.D., Fernandez D.M., Dang J.V., Santos-Molina L.F., Anzalone R.A., Garcia-Barbon C.L., Rodriguez S. (2021). Tau Pathology Profile Across a Parietal-Hippocampal Brain Network Is Associated with Spatial Reorientation Learning and Memory Performance in the 3xTg-AD Mouse. Front. Aging.

[31] Lewis J., Dickson D.W., Lin W.L., Chisholm L., Corral A., Jones G., Yen S.H., Sahara N., Skipper L., Yager D. (2001). Enhanced neurofibrillary degeneration in transgenic mice expressing mutant tau and APP. Science.

[32] Amar F., Sherman M.A., Rush T., Larson M., Boyle G., Chang L., Götz J., Buisson A., Lesné S.E. (2017). The amyloid-β oligomer Aβ*56 induces specific alterations in neuronal signaling that lead to tau phosphorylation and aggregation. Sci. Signal..

[33] Vergara C., Houben S., Suain V., Yilmaz Z., De Decker R., Vanden Dries V., Boom A., Mansour S., Leroy K., Ando K. (2019). Amyloid-β pathology enhances pathological fibrillary tau seeding induced by Alzheimer PHF in vivo. Acta Neuropathol..

[34] Fá M., Puzzo D., Piacentini R., Staniszewski A., Zhang H., Baltrons M.A., Li Puma D.D., Chatterjee I., Li J., Saeed F. (2016). Extracellular Tau Oligomers Produce An Immediate Impairment of LTP and Memory. Sci. Rep..

[35] Gulisano W., Maugeri D., Baltrons M.A., Fà M., Amato A., Palmeri A., D’Adamio L., Grassi C., Devanand D.P., Honig L.S. (2018). Role of Amyloid-β and Tau Proteins in Alzheimer’s Disease: Confuting the Amyloid Cascade. J. Alzheimer′s. Dis..

[36] Grammas P., Ovase R. (2001). Inflammatory factors are elevated in brain microvessels in Alzheimer’s disease. Neurobiol. Aging.

[37] Patel N.S., Paris D., Mathura V., Quadros A.N., Crawford F.C., Mullan M.J. (2005). Inflammatory cytokine levels correlate with amyloid load in transgenic mouse models of Alzheimer’s disease. J. Neuroinflamm..

[38] Heneka M.T., Kummer M.P., Stutz A., Delekate A., Schwartz S., Vieira-Saecker A., Griep A., Axt D., Remus A., Tzeng T.C. (2013). NLRP3 is activated in Alzheimer’s disease and contributes to pathology in APP/PS1 mice. Nature.

[39] He P., Zhong Z., Lindholm K., Berning L., Lee W., Lemere C., Staufenbiel M., Li R., Shen Y. (2007). Deletion of tumor necrosis factor death receptor inhibits amyloid beta generation and prevents learning and memory deficits in Alzheimer’s mice. J. Cell Biol..

[40] Yang L., Zhou R., Tong Y., Chen P., Shen Y., Miao S., Liu X. (2020). Neuroprotection by dihydrotestosterone in LPS-induced neuroinflammation. Neurobiol. Dis..

[41] Laurent C., Dorothée G., Hunot S., Martin E., Monnet Y., Duchamp M., Dong Y., Légeron F.P., Leboucher A., Burnouf S. (2017). Hippocampal T cell infiltration promotes neuroinflammation and cognitive decline in a mouse model of tauopathy. Brain.

[42] Terada T., Yokokura M., Obi T., Bunai T., Yoshikawa E., Ando I., Shimada H., Suhara T., Higuchi M., Ouchi Y. (2019). In vivo direct relation of tau pathology with neuroinflammation in early Alzheimer’s disease. J. Neurol..

[43] Zhao Y., Tan S.W., Huang Z.Z., Shan F.B., Li P., Ning Y.L., Ye S.Y., Zhao Z.A., Du H., Xiong R.P. (2021). NLRP3 Inflammasome-Dependent Increases in High Mobility Group Box 1 Involved in the Cognitive Dysfunction Caused by Tau-Overexpression. Front. Aging Neurosci..

[44] Kaltschmidt B., Uherek M., Volk B., Baeuerle P.A., Kaltschmidt C. (1997). Transcription factor NF-kappaB is activated in primary neurons by amyloid beta peptides and in neurons surrounding early plaques from patients with Alzheimer disease. Proc. Natl. Acad. Sci. USA.

[45] Xie L., Zhang N., Zhang Q., Li C., Sandhu A.F., Iii G.W., Lin S., Lv P., Liu Y., Wu Q. (2020). Inflammatory factors and amyloid β-induced microglial polarization promote inflammatory crosstalk with astrocytes. Aging.

[46] Sun J., Qin X., Zhang X., Wang Q., Zhang W., Wang M. (2021). FBXW11 deletion alleviates Alzheimer’s disease by reducing neuroinflammation and amyloid-β plaque formation via repression of ASK1 signaling. Biochem. Biophys. Res. Commun..

[47] Wang Q., Walsh D.M., Rowan M.J., Selkoe D.J., Anwyl R. (2004). Block of long-term potentiation by naturally secreted and synthetic amyloid beta-peptide in hippocampal slices is mediated via activation of the kinases c-Jun N-terminal kinase, cyclin-dependent kinase 5, and p38 mitogen-activated protein kinase as well as metabotropic glutamate receptor type 5. J. Neurosci..

[48] Schnöder L., Gasparoni G., Nordström K., Schottek A., Tomic I., Christmann A., Schäfer K.H., Menger M.D., Walter J., Fassbender K. (2020). Neuronal deficiency of p38α-MAPK ameliorates symptoms and pathology of APP or Tau-transgenic Alzheimer’s mouse models. FASEB J..

[49] Ruganzu J.B., Peng X., He Y., Wu X., Zheng Q., Ding B., Lin C., Guo H., Yang Z., Zhang X. (2022). Downregulation of TREM2 expression exacerbates neuroinflammatory responses through TLR4-mediated MAPK signaling pathway in a transgenic mouse model of Alzheimer’s disease. Mol. Immunol..

[50] Saha R.N., Jana M., Pahan K. (2007). MAPK p38 regulates transcriptional activity of NF-kappaB in primary human astrocytes via acetylation of p65. J. Immunol..

[51] Vanden Berghe W., Plaisance S., Boone E., De Bosscher K., Schmitz M.L., Fiers W., Haegeman G. (1998). p38 and extracellular signal-regulated kinase mitogen-activated protein kinase pathways are required for nuclear factor-kappaB p65 transactivation mediated by tumor necrosis factor. J. Biol. Chem..

[52] Zhang H.J., Wang X.Z., Cao Q., Gong G.H., Quan Z.S. (2017). Design, synthesis, anti-inflammatory activity, and molecular docking studies of perimidine derivatives containing triazole. Bioorg. Med. Chem. Lett..

[53] Zhi T.X., Liu K.Q., Cai K.Y., Zhao Y.C., Li Z.W., Wang X., He X.H., Sun X.Y. (2022). Anti-Lung Cancer Activities of 1,2,3-Triazole Curcumin Derivatives via Regulation of the MAPK/NF-κB/STAT3 Signaling Pathways. ChemMedChem.

[54] An F.M., Liu Z., Xuan X.R., Liu Q.S., Wei C.X. (2021). Sanweidoukou decoction, a Chinese herbal formula, ameliorates β-amyloid protein-induced neuronal insult via modulating MAPK/NF-κB signaling pathways: Studies in vivo and in vitro. J. Ethnopharmacol..

[55] Bruszt N., Bali Z.K., Tadepalli S.A., Nagy L.V., Hernádi I. (2021). Potentiation of cognitive enhancer effects of Alzheimer’s disease medication memantine by alpha7 nicotinic acetylcholine receptor agonist PHA-543613 in the Morris water maze task. Psychopharmacology.

